# Structural and functional insights into Uly1040, an ulvan lyase from polysaccharide lyase family 40

**DOI:** 10.1128/aem.02101-25

**Published:** 2026-01-14

**Authors:** Hou-Qi Wang, Chuan-Lei Suo, Dan Liu, Meng-Qi Wang, Jian-Xun Li, Hai-Yan Cao, Qi-Long Qin, Yu-Zhong Zhang, Peng Wang, Fei Xu

**Affiliations:** 1 MOE Key Laboratory of Evolution and Marine Biodiversity, State Key Laboratory of Marine Food Processing and Safety Control, Frontiers Science Center for Deep Ocean Multispheres and Earth System & College of Marine Life Sciences, Ocean University of China 506915 , Qingdao, Shandong, China; 2 Marine Biotechnology Research Center, State Key Laboratory of Microbial Technology, Shandong University 520252, Qingdao, China; 3 Laboratory for Marine Biology and Biotechnology, Qingdao Marine Science and Technology Center 554912 , Qingdao, China; Georgia Institute of Technology, Atlanta, Georgia, USA

**Keywords:** marine algal polysaccharide, ulvan lyase, polysaccharide lyase 40 family, catalytic mechanism

## Abstract

Ulvan is a major polysaccharide in marine green algae. Its oligosaccharide degradation products possess diverse bioactivities and hold considerable potential for various applications. Ulvan lyases, the key enzymes responsible for cleaving ulvan glycosidic bonds, generate bioactive oligosaccharides and play an essential role in ulvan degradation. However, studies on ulvan lyases remain limited, particularly for the poorly characterized polysaccharide lyase (PL) 40 family. Here, we identified Uly1040, a novel PL40 ulvan lyase, from the marine bacterium *Alteromonas macleodii* isolated from the intestine of an *Aplysia* sea slug. Uly1040 displays a unique two-domain architecture not previously reported in ulvan lyases. Mechanistically, Uly1040 employs a distinct His/Tyr catalytic strategy, divergent from known ulvan lyase mechanisms. During catalysis, Trp246 and Asn245 neutralize the negative charge of the carboxyl group at the +1 subsite. Concurrently, Mn^2+^, His487, and Asp358 activate His485 to serve as the catalytic base, while Tyr305 functions as the catalytic acid. Bioinformatic, phylogenetic, and biogeographic analyses further demonstrated that this catalytic mechanism is conserved across PL40 lyases and that Uly1040-like enzymes are widespread in marine environments. Collectively, these findings expand our understanding of PL40 ulvan lyases and provide new insights into the enzymatic basis of marine biomass utilization.

**IMPORTANCE:**

Ulvan is a major structural polysaccharide in marine green algae, and its enzymatic degradation releases bioactive oligosaccharides with promising biotechnological potential. Ulvan lyases are key to this process, yet most characterized enzymes belong to only a few polysaccharide lyase families, leaving the PL40 family largely unexplored. Here, we identify and characterize Uly1040, a novel PL40 ulvan lyase from Alteromonas macleodii, revealing an unprecedented two-domain architecture and a distinct His/Tyr catalytic mechanism. Structural and biochemical analyses show that Mn2+, His487, and Asp358 cooperatively activate His485 as the catalytic base, while Tyr305 acts as the catalytic acid-representing a mechanistic innovation in ulvan cleavage. Bioinformatic and phylogenetic analyses indicate that the PL40 lyases are widespread in marine environment, and this catalytic strategy is conserved among PL40 enzymes. This work uncovers a previously unknown enzymatic paradigm for ulvan degradation, deepening our understanding of marine polysaccharide utilization and microbial carbon cycling.

## INTRODUCTION

Ulvan is the major cell wall component of the marine green alga *Ulva*, one of the key contributors to green tides (1–6). It accounts for 9%–29% of algal dry weight, making it a significant carbon source in marine ecosystems (7). Ulvan is a sulfated complex polysaccharide, predominantly composed of 3-sulfated rhamnose (Rha3S), xylose (Xyl), glucuronic acid (GlcA), and iduronic acid (IdoA) (7). Structurally, these monosaccharides form three types of repeating disaccharide units: Rha3S-GlcA, Rha3S-IdoA, and Rha3S-Xyl arranged randomly within the polymer (8–10). Different sources of ulvan exhibit variations in monosaccharide composition and ratios. For example, Qi et al. extracted ulvan from *U. clathrata* with only 10.7 mol% Rha3S (11), whereas Cho extracted ulvan from *U. prolifera*, which demonstrated 87.6 mol% Rha3S (12). The degradation products of ulvan, termed ulvan oligosaccharides (UOS), have been shown to exhibit a variety of biological activities, including immunomodulatory (13, 14), antioxidant (15, 16), anticarcinogenic (17), anticoagulant, antihyperlipidemic (18, 19), and antiviral properties (20, 21), which are attributed to its abundant sulfated groups and uronic acid content. These attributes position ulvan as a promising candidate for applications in biomedicine, agriculture, food, and cosmetics (22). However, the resistance of ulvan’s long-chain polysaccharides to degradation hinders its utilization. Consequently, enzymes capable of degrading ulvan are of considerable biotechnological interest and have potential applications in both environmental and industrial contexts.

Ulvan lyases are enzymes that degrade ulvan, playing key roles in the initial enzymatic degradation of this sulfated polysaccharide, producing UOS (23, 24). These lyases cleave the 1,4 O-glycosidic linkage between Rha3S and GlcA or IdoA through a β-elimination mechanism, resulting in the formation of unsaturated 4-deoxy-L-*threo*-hex-4-enopyranosiduronic acid (Δ) at the non-reducing end (23, 25, 26). In the CAZy database, the currently known ulvan lyases are distributed into five polysaccharide lyase (PL) families (PL24, PL25, PL28, PL37, and PL40), sourced from six bacterial genera across two phyla (Pseudomonas and Bacteroidota) (25–38). Despite their significant ecological significances and biotechnological potentials, structural and mechanistic studies remain limited. To date, only three ulvan lyases from the PL24, PL25, and PL28 families have been structurally resolved, revealing two distinct structural folds, a seven-bladed β-propeller and a β-sandwich jelly-roll fold (30, 35, 36, 39). The PL40 family ulvan lyase is particularly understudied, with only one member (the enzyme P10_PLnc from *Formosa agariphila*) functionally characterized (24).

In this study, a novel PL40 ulvan lyase, Uly1040, from a ulvan-degrading strain *Alteromonas macleodii* strain HT-3, was uncovered. Uly1040 is a 97 kDa protein that exhibits endolytic activity, cleaving ulvan polysaccharides primarily into unsaturated disaccharides. Its crystal structure was determined at a resolution of 1.74 Å, revealing an N-terminal (α/α)_6_ toroid domain and a C-terminal anti-parallel β-sheet domain. Through structural comparison, sequence alignment, molecular docking, and biochemical validation, the catalytic mechanism of Uly1040 in ulvan cleavage was elucidated. Furthermore, bioinformatics analyses were performed to investigate the phylogenetic distribution of Uly1040-representative PL40 ulvan lyases among bacterial taxa and their biogeographic patterns, while evaluating the conservation of their catalytic mechanisms.

## RESULTS AND DISCUSSION

### Sequence analysis and purification of the new PL40 family member Uly1040

**Fig 1:**
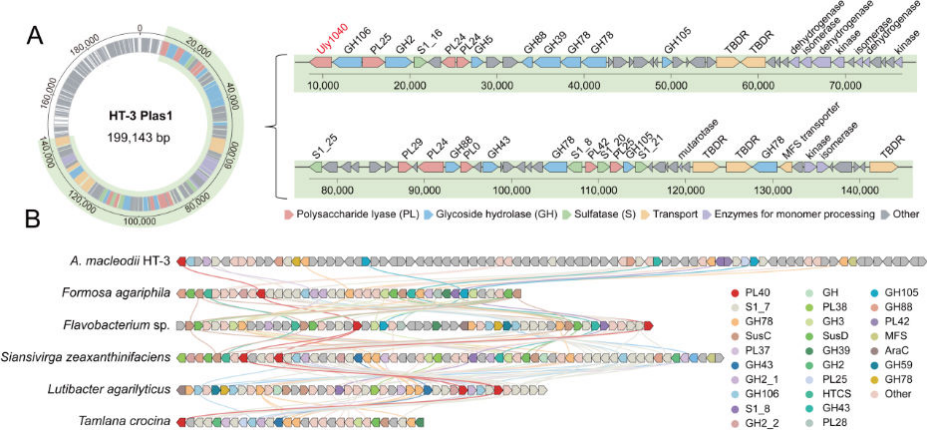
Analysis of the ulvan utilization locus (UUL) from *Alteromonas macleodii* HT-3. (**A**) Genomic location and genetic organization of the UUL from *Alteromonas macleodii* HT-3. Genes are represented as arrows colored by predicted functional categories. The UUL region is highlighted in green, encompassing genes predicted to be involved in ulvan depolymerization and utilization, including polysaccharide lyases (PLs), sulfatases (Ss), glycoside hydrolases (GHs), enzymes for monomer processing, and transporters such as TonB-dependent receptors (TBDRs) and a major facilitator superfamily transporter (MFS). (**B**) Synteny comparison of the UUL from *A. macleodii* HT-3 with the UUL from *Formosa agariphila* and five additional PL40-containing polysaccharide utilization loci from diverse Bacteroidota genera. PULs were aligned using Clinker, with homologous genes connected by color-matched lines.

*A. macleodii* strain HT-3, isolated from the intestinal tract of an *Aplysia* sea slug, was found to utilize ulvan as its sole carbon source. Whole-genome sequencing revealed that the strain carries an ulvan utilization locus (UUL) on Plasmid 1, designated UUL_HT3_, which comprises multiple proteins involved in ulvan utilization. The UUL_HT3_ includes putative ulvan lyases from families PL24, PL25, and PL40, multiple S1 family sulfatases, glycoside hydrolases (GHs), enzymes for monomer processing, and transporters such as outer-membrane TonB-dependent receptors (TBDRs) and an inner-membrane MFS transporter (Fig. 1A). These enzymes are predicted to mediate the complete ulvan utilization process: extracellular depolymerization of the ulvan backbone by ulvan lyases, subsequent desulfation by sulfatases, hydrolysis of unsaturated oligosaccharides by GHs, and final conversion of the released monosaccharides into central metabolic pathways.

Clinker analysis showed that UUL_HT3_ exhibits strong functional correspondence with the previously characterized ulvan utilization locus from *Formosa agariphila,* as well as with five other PL40-containing polysaccharide utilization loci (PULs) from various Bacteroidota genera (retrieved from PULDB; https://www.cazy.org/PULDB) (Fig. 1B) (24, 40). These loci share a conserved set of core enzymatic components, including ulvan-specific lyases, multiple sulfatases, GHs for oligosaccharide cleavage, and transport systems, indicating a convergent functional strategy for ulvan degradation despite low gene-level synteny.

**Fig 2:**
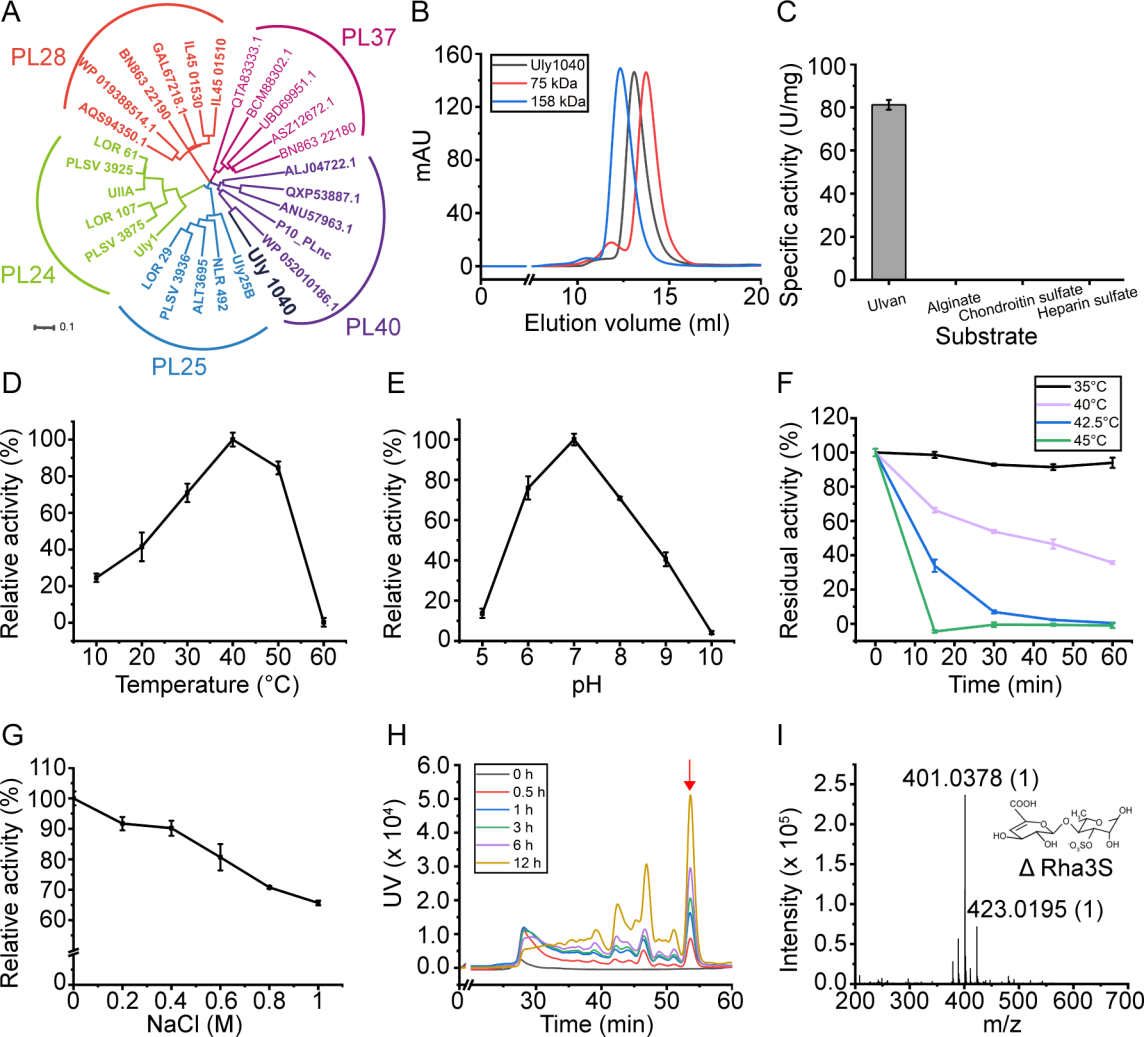
Sequence analysis and biochemical characterization of Uly1040. (**A**) Phylogenetic analysis of Uly1040 with ulvan lyases from the PL24, PL25, PL28, PL37, and PL40 families. Sequences were retrieved from the CAZy database, including all characterized ulvan lyases. The phylogenetic tree was constructed using the neighbor-joining method with a Poisson model. (**B**) Gel filtration analysis of Uly1040 in solution. Conalbumin (75 kDa) and aldolase (158 kDa) served as protein size markers. The predicted molecular mass of Uly1040 is 97 kDa. (**C**) Substrate specificity of Uly1040 toward ulvan, heparan sulfate, and alginate. (**D**) Effect of temperature on Uly1040 activity. The highest activity at 40°C was set to 100%. (**E**) Effect of pH on Uly1040 activity. Reactions were performed at 40°C in Britton-Robinson buffer (pH 5.0–10.0). The highest activity at pH 7.0 was set to 100%. (**F**) Thermal stability of Uly1040. The enzyme was incubated at 30, 40, and 50°C for 0–60 min, and residual activity was measured at 40°C and pH 7.0. The highest activity after incubation at 4°C was set to 100%. (**G**) Effect of NaCl concentration on Uly1040 activity. The activity at 0 M NaCl was set to 100%. (**H**) Time-course analysis of Uly1040 degradation products from ulvan (0–12 h). Products were analyzed by gel filtration chromatography (Superdex peptide 10/300 GL) monitored at 235 nm. The major peak (red arrow) was identified as the main product. Data in panels (**C–G**) represent mean ± standard deviation (SD) from three independent experiments. (**I**) ESI-MS analysis of the major peak from panel H. The dominant peak showed an *m*/*z* of 401, consistent with the disaccharide ΔRha3S.

Within UUL_HT3_, a novel ulvan lyase gene was discovered, spanning 2,571 base pairs and encoding an 856-amino-acid protein with a 16-residue signal peptide. This enzyme was designated Uly1040. Phylogenetic analysis placed Uly1040 within the PL40 family, showing 30.61% sequence identity to the previously reported PL40 ulvan lyase P10_PLnc (Fig. 2A).

For biochemical characterization, an expression vector for Uly1040 was constructed, and the recombinant protein was expressed and purified to homogeneity. The purified enzyme exhibited an apparent molecular weight of ~90 kDa by SDS-PAGE analysis (Fig. S1), consistent with the theoretical value of 97 kDa. Gel filtration chromatography further demonstrated that recombinant Uly1040 exists as a monomer in solution (Fig. 2B).

### Characterization of Uly1040

Given the structural similarities among sulfated polysaccharides and the reported cross-reactivity of certain polysaccharide lyases with ulvan, heparan sulfate, and chondroitin sulfate (32), we evaluated the substrate specificity of Uly1040 using these three polysaccharides, along with alginate, a common algal polysaccharide substrate for many polysaccharide lyases. Uly1040 exhibited activity exclusively toward ulvan and showed no detectable activity against heparan sulfate, chondroitin sulfate, or alginate (Fig. 2C), demonstrating its strict specificity as an ulvan lyase.

The enzymatic properties of Uly1040 were then examined. The enzyme displayed optimal activity at 40°C (Fig. 2D) and pH 7.0 (Fig. 2E). Thermal stability assays revealed that Uly1040 retained full activity after 1 h at 35°C but was rapidly inactivated at 45°C, losing nearly all activity within 15 min (Fig. 2F). Uly1040 activity was also sensitive to NaCl concentration, showing a progressive decline from 0 to 1.0 M NaCl (Fig. 2G). Unlike most ulvan lyases derived from marine environments, which typically show maximal activity at 0.5–1.0 M NaCl, Uly1040 is salt-sensitive, likely reflecting adaptation to the lower-salinity intestinal environment of its sea slug host.

To elucidate its mode of action, we performed a time-course analysis of ulvan degradation under optimal conditions. Chromatographic profiles revealed three major product peaks accompanied by several minor ones. While the peak intensities increased over time, the number of distinct peaks remained unchanged (Fig. 2H), consistent with an endolytic cleavage mechanism that produces low-molecular-weight oligosaccharides. The predominant product (*m*/*z* 401) was identified by negative electrospray mass spectrometry as the disaccharide ΔRha3S (Fig. 2I), which consists of a 3-sulfated rhamnose (Rha3S) linked to the Δ. This confirms that ΔRha3S is the principal degradation product. Such a product profile is consistent with other ulvan lyases, which typically yield disaccharides as the major products, accompanied by tetrasaccharides and oligosaccharides with higher degrees of polymerization (26).

### Overall structure of Uly1040

**Fig 3:**
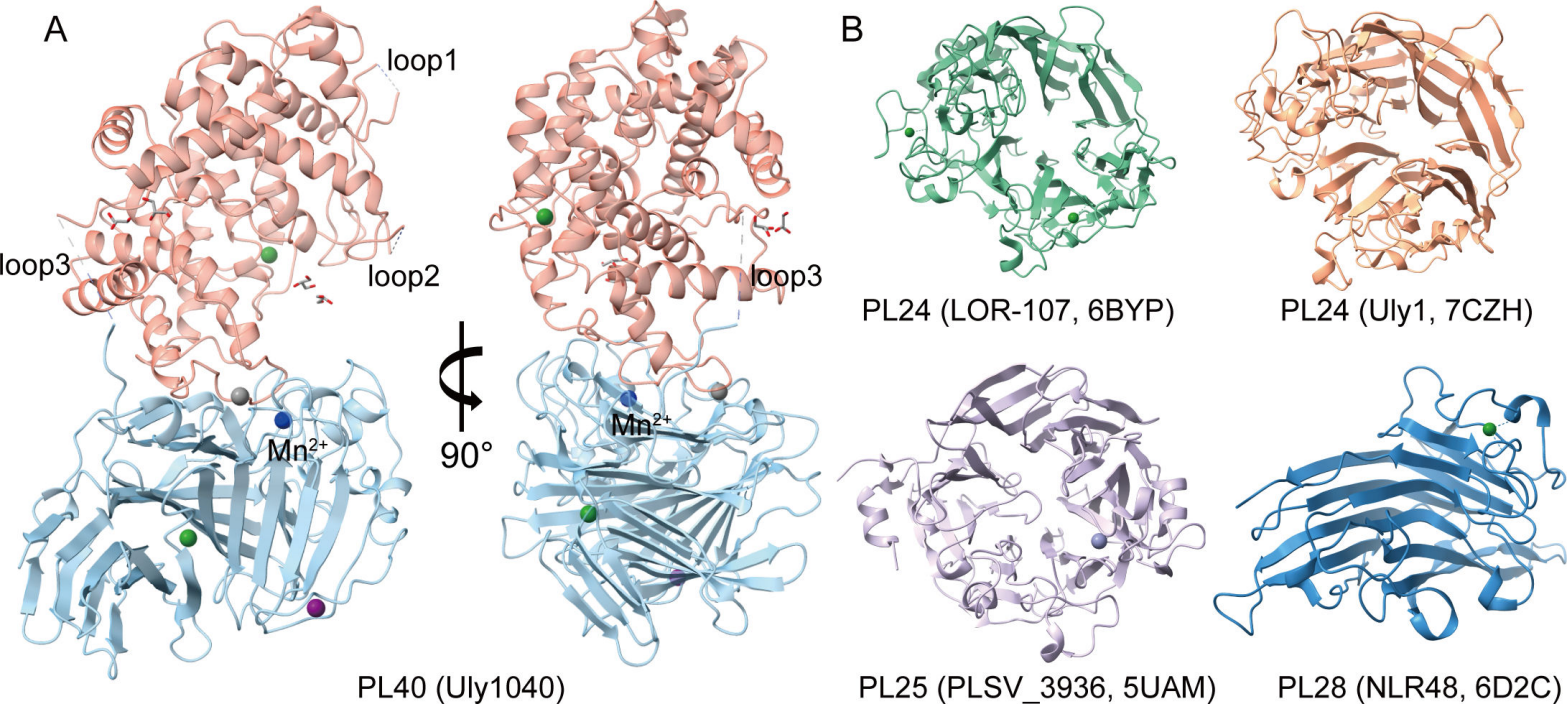
Overall structure of Uly1040 and structural comparison with ulvan lyases from other PL families. (**A**) Overall structure of Uly1040. The N- and C-terminal domains are shown in light pink and light blue, respectively. Ca^2+^, Mn^2+^, Fe^3+^, and Mg^2+^ ions are represented as green, blue, gray, and purple spheres, respectively. (**B**) The structures of ulvan lyases from other PL families. PL24 lyase LOR-107 (PDB: 6BYP) is shown in green, PL24 lyase Uly1 (PDB: 7CZH) in orange, PL25 lyase PLSV_3936 (PDB: 5UAM) in purple, and PL28 lyase NLR48 (PDB: 6D2C) in blue.

To elucidate the catalytic mechanism of Uly1040, we determined its crystal structure at 1.74 Å resolution. The protein consists of two distinct domains: an N-terminal (α/α)_6_ toroid domain (residues 27–430) and a C-terminal antiparallel β-sheet domain (residues 444–856), connected by a short linker (residues 431–443) (Fig. 3A). The N-terminal domain contains 16 α-helices and 2 β-strands, with 12 helices contributing to the (α/α)_6_ toroid fold. The C-terminal domain is composed of 7 α-helices and 29 β-strands, which assemble into six antiparallel β-sheets. Three loops (loop1, residues 85–91; loop2, residues 102–109; loop3, residues 431–443) were not resolved in the electron density, most likely due to conformational flexibility (Fig. 3A). In addition, four glycerol molecules were observed, presumably incorporated during crystal soaking in glycerol-containing cryoprotectant solution.

The structure of Uly1040 is distinct from all previously characterized ulvan lyases. For example, LOR-107 and Uly1 of PL24 and PLSV3936 of PL25 adopt a seven-bladed β-propeller fold (31, 36, 40), whereas NLR48 of PL28 exhibits a β-jelly-roll fold (Fig. 3B) (37). In contrast, structural comparison using the Dali server revealed that Uly1040 resembles the PL12 heparan-sulfate lyase Phep_3797 (PDB: 4MMI), the PL15 exo-heparin lyase BIexoHep (PDB: 6LJA), the PL17 alginate lyase Alg17C (PDB: 4NEI), and the PL39 alginate lyase Dp0100 (PDB: 6JPN) (Table S1) (41). These enzymes share a common architecture comprising an (α/α)_n_ barrel-type toroidal fold domain and an antiparallel β-sheet domain (42–45), with catalytic cavities located at the domain interface, suggesting a similar arrangement in Uly1040. Collectively, these findings indicate that Uly1040 defines a previously unrecognized structural architecture within ulvan lyases.

The electron density map revealed five distinct peaks in each asymmetric unit, each presumed to correspond to a bound metal ion. Inductively coupled plasma optical emission spectrometry (ICP-OES) analysis of the purified Uly1040 confirmed the presence of Mn^2+^, Fe^3+^, Mg^2+^, and Ca^2+^ (Table S2). Among these, a metal ion situated near the potential catalytic cavity is proposed to be involved in catalysis. Based on its coordination geometry and bond lengths, it was assigned as Mn^2+^, an identification further supported by the CheckMyMetal server (Fig. S2; Table S3) (46). The remaining four metal ions were tentatively modeled as Fe^3+^, Mg^2+^, and Ca^2+^ though these assignments require further validation.

### Key residues involved in the catalytic activity of Uly1040

**Fig 4:**
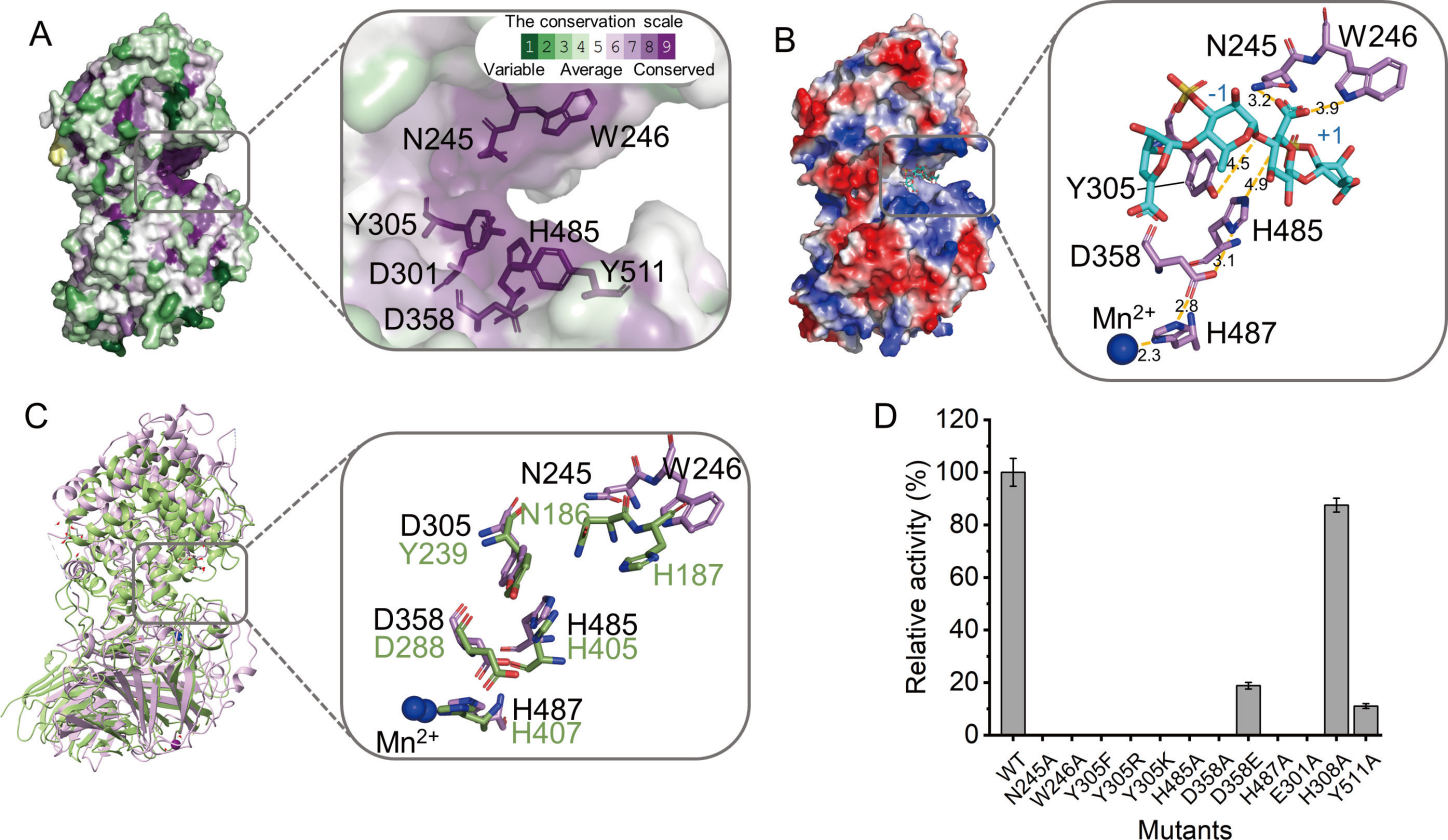
Key amino acid residues in the active site of Uly1040. (**A**) ConSurf analysis of Uly1040. The color scale denotes conservation levels, from 1 (variable, turquoise) to 9 (highly conserved, purple). (**B**) Key amino acid residues involved in catalysis and charge neutralization in the Uly1040-tetrasaccharide complex. The protein surface is colored by electrostatic potential (red for negative, blue for positive). The docked tetrasaccharide (Δ-Rha3S-GlcA-Rha3S) is shown as blue sticks, with surrounding residues shown as purple sticks. Distances are indicated by yellow dashed lines. (**C**) Structural alignment of the active-site residues in Uly1040 and the PL39 alginate lyase Dp0100. Uly1040 is shown in purple, Dp0100 in forest green, and Mn^2+^ ions as blue spheres. (**D**) Enzymatic activity of Uly1040 mutants toward ulvan. Activity of the wild type (WT) was set to 100%. Data represent mean ± standard deviation (SD) from three independent experiments.

Structural comparisons suggest that Uly1040 harbors a catalytic cavity at the interface between its N- and C-terminal domains. Consurf analysis of Uly1040 and its homologs revealed two clusters of conserved polar residues at this interface, including Asn245, Trp246, Asp301, Tyr305, and Asp358 in the N-terminal domain and His485 and Tyr511 in the C-terminal domain (Fig. 4A) (47). To probe their roles, we attempted to crystallize Uly1040 in complex with either UOS or the disaccharide product; however, no complex structures could be obtained. Consequently, docking simulation was performed using a ulvan tetrasaccharide (Δ-Rha3S-GlcA-Rha3S) (48, 49). The tetrasaccharide was positioned within the interdomain cavity, which is open at both ends, consistent with Uly1040’s endolytic cleavage pattern (Fig. 4B). The positively charged surface of the cavity likely facilitates binding to the polyanionic ulvan chain, while the 1,4 O-glycosidic linkage between Rha3S and GlcA represents the cleavage site, with the tetrasaccharide spanning subsites −2 to +2 from the nonreducing to the reducing terminus.

The β-elimination mechanism requires neutralization of the carboxyl group at the +1 subsite (GlcA). Two conserved residues, Asn245 and Trp246, interact with this group (Fig. 4B; Fig. S3). Among them, Asn245 is positioned closer to the group (3.2 Å) compared to Trp246 (3.9 Å). Alanine substitution of either residue abolished activity toward ulvan (Fig. 4D), suggesting that Asn245 primarily mediates charge neutralization, while Trp246 contributes via hydrogen bonding between its amide group and the C-6 carboxylate oxygen.

For the catalytic acid and base, His485 is positioned near the C-5 atom of the +1 glycuronate (4.9 Å), and Tyr305 is adjacent to the glycosidic oxygen bridging the −1 and +1 sugars (4.5 Å) (Fig. 4B). Mutation of His485 to alanine or Tyr305 to phenylalanine abolished enzymatic activity, and substitution of Tyr305 with basic residues (Y305K or Y305R) also eliminated activity, confirming that His485 acts as the catalytic base and Tyr305 as the catalytic acid. Structural analysis of the active site revealed that the ND1 atom of His485 is 3.1 Å from the OD2 atom of Asp358, which is 2.8 Å from His487 coordinating a putative Mn^2+^ ion (2.3 Å distance). This hydrogen-bond network likely activates His485 by lowering its p*K*_a_ and enhancing nucleophilicity. Mutation of Asp358 or His487 to alanine completely inactivated the enzyme, whereas D358E retained ~18% of wild-type (WT) activity, further supporting His485’s role as the catalytic base. A similar tetrad arrangement is observed in Dp0100 (His405-Asp288-His407-Mn^2+^), suggesting that tetrad-assisted activation of the catalytic base is a conserved mechanistic feature between PL40 ulvan lyases and PL39 alginate lyases (Fig. 4C) (45).

Additional conserved residues, Glu301 and Tyr511, likely contribute to substrate recognition, while polar non-conserved residues His308 appear non-essential (Fig. S3 and S4). Mutations E301A and Y511A significantly reduced activity, whereas H308A showed minimal effect (Fig. 4D). Circular dichroism (CD) spectra confirmed that all mutants retained secondary structure comparable to WT Uly1040, indicating that the observed changes in activity reflect specific residue functions rather than global structural perturbations (Fig. 4D; Fig. S5).

### Proposed catalytic mechanism of Uly1040

Polysaccharide lyase catalysis generally involves three steps: (i) stabilization or neutralization of the negative charge on the C-6 carboxylate anion, (ii) abstraction of the C-5 proton by the catalytic base, and (iii) proton donation by the catalytic acid to cleave the glycosidic bond (50).

**Fig 5:**
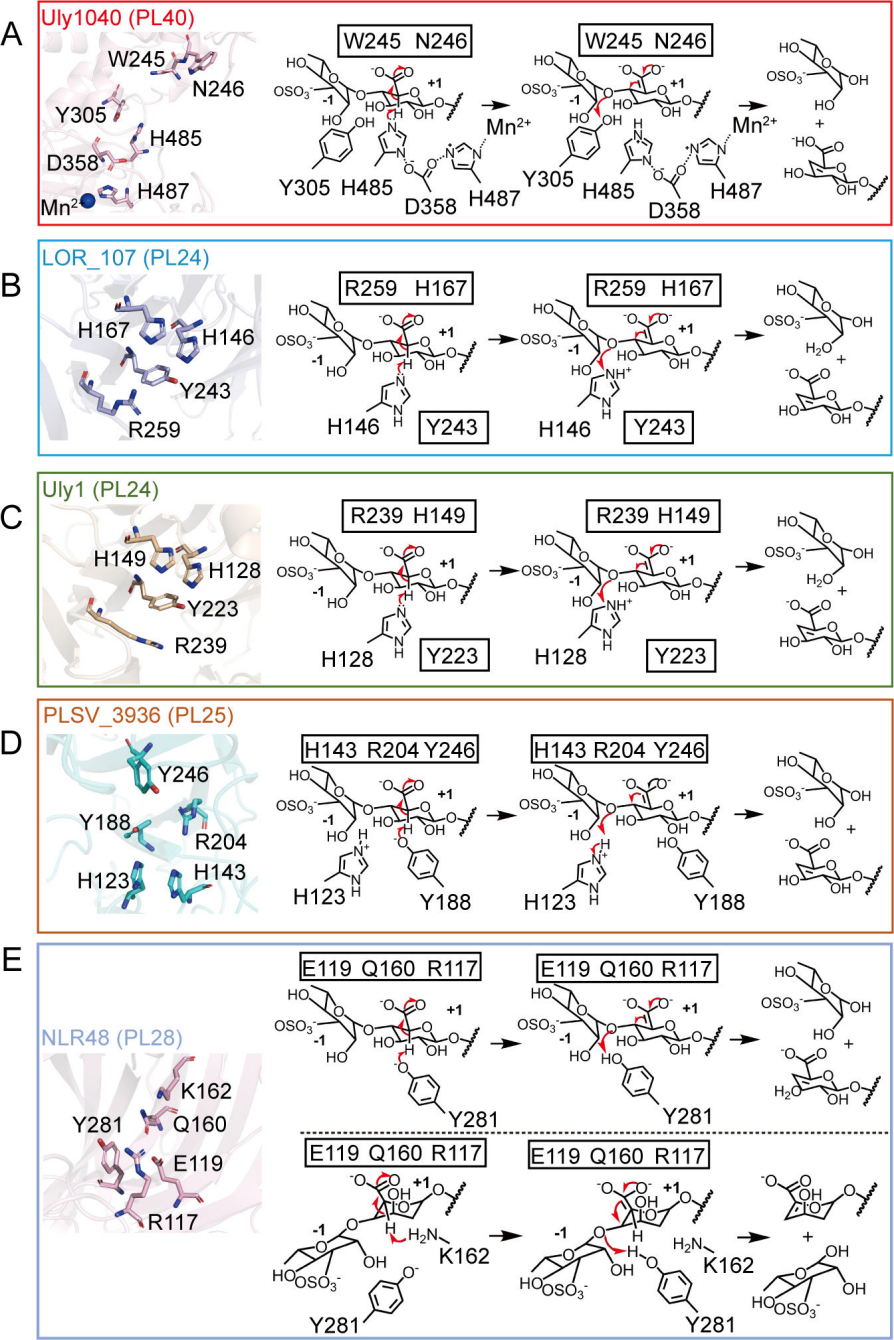
Catalytic mechanism of Uly1040 and comparison with ulvan lyases from other families. (**A**) Schematic representation of the catalytic residues and proposed catalytic mechanism of Uly1040. (**B–E**) Catalytic residue arrangements and reaction mechanisms of representative ulvan lyases: PL24 LOR_107 (**B**), PL24 Uly1 (**C**), PL25 PLSV_3936 (**D**), and PL28 NLR48 (**E**). In panel E, the upper scheme shows the cleavage mechanism when GlcA occupies the +1 subsite, whereas the lower scheme illustrates the mechanism when IdoA occupies the +1 subsite. Residues highlighted with black boxes indicate those with neutralizing or auxiliary functions.

For Uly1040, we propose the following molecular mechanism for catalysis. Upon substrate binding, residues involved in substrate recognition, such as Glu301 and Tyr511, position the ulvan chain within the catalytic cavity near the key catalytic residues. The conserved residues Asn245 and Trp246 neutralize the negative charge of the carboxyl group at the +1 subsite. Mn^2+^, His487, and Asp358 cooperatively activate His485, enabling it to function as the catalytic base and abstract the C-5 proton. Simultaneously, Tyr305 acts as the catalytic acid, donating a proton to the glycosidic oxygen to facilitate bond cleavage (Fig. 5A).

Ulvan lyases are classified into the PL24, PL25, PL28, PL37, and PL40 families (26), with structural and mechanistic analyses available for enzymes from PL24, PL25, and PL28 (Fig. 5) (30, 35, 36, 39). PL24 lyases LOR-107 and Uly1 use His for both acid/base catalysis, assisted by Arg residues and Tyr as auxiliary catalysts (35, 39). PL25 lyase PLSV_3936 employs a Tyr/His mechanism, with Arg neutralizing acidic groups (30). PL28 lyase NLR48 uses Gln for neutralization, with catalytic roles dependent on the +1 subsite: Tyr acts as both acid/base for GlcA, and Tyr/Lys function as acid/base for IdoA (Fig. 5B through E) (36).

In contrast, Uly1040 exhibits a distinct catalytic strategy. It relies on Asn245 and Trp246 for neutralization, His485 exclusively as the catalytic base, and Tyr305 as the catalytic acid. Uniquely, His485 is activated via a tetrad network involving Asp358, His487, and Mn^2+^, enhancing its nucleophilicity, a feature not observed in other ulvan lyases. This contrasts with the dual-role His in PL24, the reversed roles in PL25, and the substrate-dependent roles in PL28. Therefore, while all these enzymes target ulvan, Uly1040 defines a novel mechanistic paradigm among ulvan lyases.

### Conserved catalytic mechanism and global marine distribution of PL40 ulvan lyases

**Fig 6:**
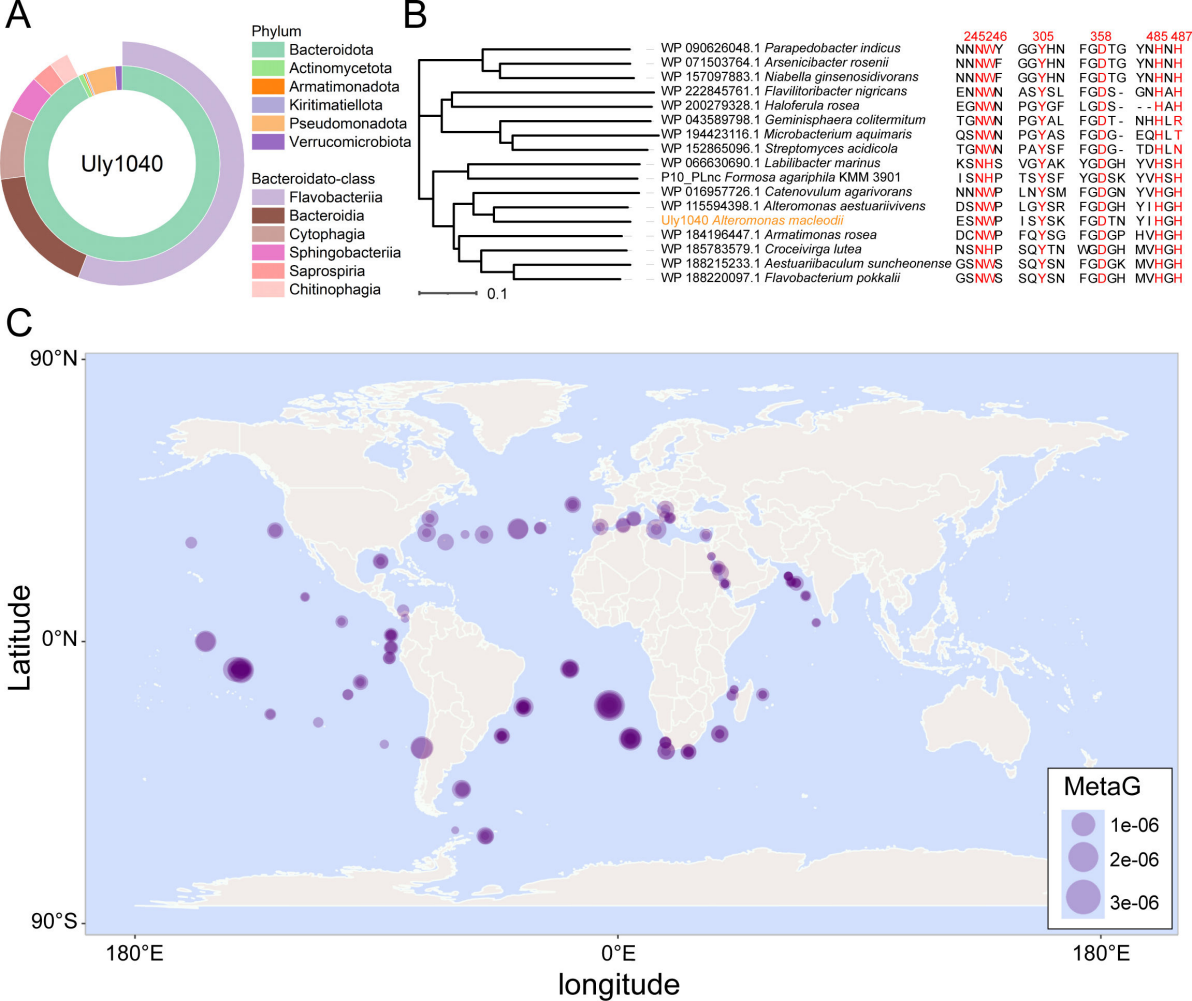
Universality of Uly1040 homologs in the marine environment. (**A**) Phylogenetic distribution of Uly1040 homologs. Sequences were retrieved from the NCBI non-redundant protein database using Uly1040 as a query, with thresholds of *E*-value < 1*e*-10 and sequence identity >30%. Distributions were analyzed at the phylum level across all taxa and at the class level within the Bacteroidota phylum. (**B**) Sequence alignment of Uly1040 homologs from diverse bacterial phyla. Phylogenetic analysis of representative strains was conducted based on 16S rRNA sequences using the neighbor-joining method in MEGA-X with 1,000 bootstrap replicates. Catalytic key residues are highlighted in red. (**C**) Geographic distribution and relative abundance of Uly1040 homologs in the ocean. Uly1040 homologs were identified in Tara Oceans metagenomic data sets (OM-RGCv1) using a homology search threshold of *E*-value < 1*e*-10. Gene abundance was normalized to mapped read percentages, with circle sizes scaled to relative abundance. World map background was generated using the R maps package (data source: CIA World Data Bank II).

To examine the conservation of catalytic mechanism within the PL40 family, homologous sequences were retrieved from the Non-Redundant Protein Sequence Database using Uly1040 as queries. All sequences are bacterial, predominantly from Bacteroidota (92.6%) with a minor fraction from Pseudomonadota (4.8%) (Fig. 6A). Within Bacteroidota, sequences are mainly from the Flavobacteriia (60.3%) and Bacteroidia (18.5%) classes. Comparison across phyla revealed that key catalytic residues of Uly1040, including Asn245, Tyr305, Asp358, and His485, are strictly conserved (Fig. 6B). Trp246, responsible for carboxyl neutralization, is occasionally replaced by His, which likely preserves function due to its amide group. His487 is also highly conserved, with only rare substitutions by Arg, Thr, or Asn observed (Fig. 6B). These results indicate that the catalytic mechanism of Uly1040 is highly conserved across the PL40 family.

Analysis using the Tara Oceans database shows that Uly1040 homologs are widely distributed in the Atlantic and eastern Pacific Oceans (Fig. 6C) (51, 52), largely consistent with the distribution of *Ulva* recorded in the GBIF database (https://www.gbif.org/), highlighting their potential role in global ulvan degradation.

### Conclusions

Ulvan is a major polysaccharide in marine green algae, contributing to oceanic biomass and coastal green tides, with its degradation products exhibiting diverse bioactivities. In this study, we characterized Uly1040, a PL40 ulvan lyase from *A. macleodii*, revealing a unique two-domain architecture and a distinct His/Tyr catalytic mechanism. Phylogenetic and biogeographic analyses indicate that Uly1040 homologs are widely distributed among the ocean, highlighting their ecological significance in ulvan degradation and marine carbon cycling. These findings provide the first mechanistic insights into the PL40 family, establishing Uly1040 as a representative model for understanding this group of ulvan lyases.

## MATERIALS AND METHODS

### Bioinformatics analysis

The genomic DNA of *A. macleodii* strain HT-3 was shotgun-sequenced on the Illumina HiSeq sequencing platform (Majorbio, China). The putative ulvan lyases in strain HT-3 were predicted by the dbCAN meta server (https://bcb.unl.edu/dbCAN2/blast.php) based on the strain’s genome (53). Synteny of PULs was analyzed using Clinker (version 0.0.31) (https://github.com/gamcil/clinker?tab=readme-ov-file) under default settings and a minimum sequence identity threshold of 30%. Homology relationships were visualized using Clinker’s built-in plotting module, which connects homologous genes based on amino acid sequence similarity.

The signal peptide regions were predicted by the SignalP 5.0 server (https://services.healthtech.dtu.dk/services/SignalP-5.0/). The phylogenetic trees were generated using MEGA-X based on multiple sequence alignment of Uly1040 with other ulvan lyases from the CAZy database (http://www.cazy.org/) (54), followed by visualization using the iTOL platform (55).

The amino acid sequence of Uly1040 (GenBank: MGY0314104.1) was used as a query to search for homologous proteins using BLASTp against the NCBI non-redundant protein sequence database with an *E*-value < 1*e*-10 and >30% sequence identity requirement. Identified homologous sequences were taxonomically classified at both phylum and class levels. Representative sequences from distinct taxonomic groups were selected for multiple sequence alignment using ClustalW (https://www.genome.jp/tools-bin/clustalw). The distribution of Uly1040 homologs in marine environments was investigated using the metagenomic data from the Tara Oceans database with an E-value threshold of 1e-10 (51, 52).

### Gene cloning

The gene encoding Uly1040 without the signal peptide was amplified from the genome of strain HT-3 and cloned into the pET-22b expression vector containing a C-terminal 6×His tag. Site-directed mutations in Uly1040 were conducted with the plasmid pET22b-Uly1040 as the template by using a QuikChange kit (Agilent Technologies, USA).

### Protein expression and purification

Recombinant proteins of Uly1040 and its mutants were overexpressed in *Escherichia coli* BL21 (DE3) (Vazyme, China) and cultured at 16°C for 14 h in lysogeny broth containing 100 μg/mL ampicillin under the induction of 0.4 mM isopropyl β-D-thiogalactopyranoside (IPTG). The recombinant proteins were purified by Ni-NTA affinity resin (Qiagen, Germany) and then fractionated by gel filtration on a Superdex 200 Increase 10/300 GL column (GE Healthcare, USA). Aldolase (158 kDa) and conalbumin (75 kDa) from GE Healthcare were used as molecular weight standards.

### Biochemical characterization

Protein concentration was determined with bovine serum albumin as the standard by using a BCA protein assay kit (Thermo Fisher Scientific, USA). The activities of WT Uly1040 and its mutants toward ulvan were measured by UV spectrophotometric method. Briefly, a 300 μL mixture containing 2.5 μg enzyme and 2 mg/mL substrate in 50 mM Tris-HCl (pH 7.0) was incubated at 40°C for 10 min. Then, the reaction mixture was boiled for 10 min to terminate the reaction, followed by centrifugation to remove the precipitate. The increase in absorbance at 235 nm (*A*_235_), resulting from the formation of unsaturated uronic acids in the mixture, was subsequently monitored. One unit of enzyme activity was defined as the amount of enzyme needed to produce an *A*_235_ increase of 0.1 per min under the assay conditions. All assays were performed at least in triplicate and results are reported as mean ± standard deviation (SD).

The optimum temperature for Uly1040 activity was determined over a range of 10 to 60°C at pH 7.0. The optimum pH for Uly1040 activity was determined at 40°C in Britton-Robinson (B-R) buffer ranging from pH 5.0 to 12.0. To evaluate thermal stability, Uly1040 was incubated at 35, 40, 42.5, and 45°C for durations ranging from 0 to 60 min. After each incubation period, the residual enzymatic activity was measured under optimal conditions (40°C, pH 7.0). The effect of NaCl on Uly1040 activity was determined at NaCl concentrations ranging from 0 to 1.0 M. The effects of selected metal ions and EDTA on Uly1040 activity were examined at pH 7.0 and 40°C at a final concentration of 0.25 or 1 mM.

The action mode and degradation products of Uly1040 were analyzed by using ulvan as the substrate. The degradation reaction was carried out at 40°C for 0 to 12 h. The ulvan degradation products were analyzed by gel filtration chromatography on a Superdex Peptide 10/300 GL column (GE Healthcare, USA) at a flow rate of 0.3 mL/min using 0.2 M ammonium hydrogen carbonate as the mobile phase. Elution was monitored at 235 nm using a UV detector. The major peak was further identified by ESI-MS on an ion trap time of flight (TOF) hybrid mass spectrometer (LCMS-IT-TOF; Shimadzu, Japan). ESI-MS analysis was set in the negative-ion mode and with the following parameters: source voltage at 3.6 kV, nebulizer nitrogen gas flow rate at 1.5 L/min, heat block, curved desolvation line temperature at 200°C, and detector voltage at 1.8 kV. The mass acquisition range was set at 200 to 800.

### Crystallization and data collection

The crystals of WT Uly1040 were obtained at 18°C using the sitting-drop vapor diffusion method with a reservoir solution containing 200 mM sodium bromide, 100 mM Bis-Tris propane (pH 8.5), and 20% (wt/vol) polyethylene glycol (PEG) 3350. All the X-ray diffraction data were collected on the BL17U1 beamline at the Shanghai Synchrotron Radiation Facility using an ADSC Quantum 315r detector. The initial diffraction data sets were processed by the HKL-2000 program. Relevant data collection statistics are shown in Table 1.

**TABLE 1:** Diffraction data and refinement statistics of Uly1040

Parameter	Value for Uly1040
Data collection	
Space group	P 1 2_1_ 1
Unit cell^*a*^	
*a* (Å)	49.02
*b* (Å)	125.90
*c* (Å)	69.74
*α* (°)	90.00
*β* (°)	97.92
*γ* (°)	90.00
Wavelength (Å)	0.9791
Resolution (Å)	48.56–1.74 (1.79–1.74)
Redundancy	6.7 (6.3)
Completeness (%)	99.1 (98.1)
*R*_merge_*^b^*	0.14 (1.48)
*I*/*σ*	6.80 (1.60)
Refinement statistics	
Resolution (Å)	37.35–1.74
*R*_work_ (%)	18.31
*R*_free_ (%)	22.69
B-factor (Å^2^)	
Protein	24.61
Solvent	32.30
Ligands	44.61
RMSD from ideal geometry	
Length (Å)	0.012
Angles (°)	1.14
Ramachandran plot (%)^*c*^	
Favored	96.0
Allowed	4.0

^*a*^ Numbers in parentheses refer to data in the highest resolution shell.

^*b*^ *R*_merge_ =Σ*_hkl_*Σ*_i_*|*I*(*hkl*)*_i_* − <*I*(*hkl*)>|/Σ*_hkl_*Σ*_i_* <*I*(*hkl*)*_i_*>.

^*c*^ The Ramachandran plot was calculated by the PROCHECK program in the CCP4i program package.

The phases were determined using the molecular replacement method (EPMR) and the CCP4 program Phaser (56). The crystal structure of Uly1040 was solved by molecular replacement using the structure predicted by AlphaFold2 as the search model (57). The structure refinement was performed using Coot and Phenix (58, 59). The quality of the final model is summarized in Table 1.

### Metal ion detection

Metal ion content in Uly1040 was quantitatively determined by ICP-OES. For sample preparation, 1 mL of purified Uly1040 protein solution was subjected to acid digestion with 10 mL of ultrapure HNO_3_ at 120°C for 12 h in a sealed digestion vessel. After evaporation to a residual volume of <1 mL, the digest was diluted to a final volume of 5 mL with triple-distilled water. The solution was then filtered through a 0.22 μm filter membrane and immediately analyzed by ICP-OES. Metal-binding sites were validated geometrically using the CheckMyMetal server (46).

### Molecular docking analysis

The refined model of Uly1040 was employed for molecular docking using AutoDock v4.2.6 (48, 49) with default parameters. The ulvan tetrasaccharide structural data were extracted from the complex structure of LOR107 R320 mutant with a tetrasaccharide substrate (PDB: 6BYT). The docking results were evaluated, and the most reasonable binding mode with the lowest binding energy was selected. Protein structure representations were prepared with PyMOL and ChimeraX.

### CD spectroscopic assay

CD spectra of Uly1040 and all mutants (~10 μM in 20 mM Tris-HCl, pH 8.0, 200 mM NaCl) were recorded at 25°C on a J-810 spectropolarimeter (Jasco, Japan). CD spectra were collected from 200 nm to 250 nm at a scan speed of 200 nm/min with a bandwidth of 2 nm.

## Data Availability

The atomic coordinates and structure factors of WT Uly1040 have been deposited in the PDB database under the accession code 9VTK. The bacterial strain *Alteromonas macleodii* HT-3 is available from the Marine Culture Collection of China (MCCC) under accession number MCCC1K10327, and its whole-genome sequence has been deposited in the GenBank database under the accession number JBSSTO000000000.

## SUPPLEMENTAL MATERIAL

Supplemental material aem.02101-25-s0001.docx Figures S1 to S5; Tables S1 to S4.

ASM does not own the copyrights to Supplemental Material that may be linked to, or accessed through, an article. The authors have granted ASM a non-exclusive, world-wide license to publish the Supplemental Material files. Please contact the corresponding author directly for reuse.

## References

[1] Hu C, Qi L, Hu L, Cui T, Xing Q, He M, Wang N, Xiao Y, Sun D, Lu Y, Yuan C, Wu M, Wang C, Chen Y, Xu H, Sun L, Guo M, Wang M. 2023. Mapping Ulva prolifera green tides from space: a revisit on algorithm design and data products. Int J Appl Earth Obs Geoinf 116:103173. doi:10.1016/j.jag.2022.103173

[2] Huan L, Shi M, Wang X, Gu W, Zhang B, Liu X, Zhuo J, Wang G. 2023. Morphological characteristics and genetic diversity of floating and attached Ulva prolifera––A case study in the Yellow Sea, China. Mar Pollut Bull 195:115468. doi:10.1016/j.marpolbul.2023.115468 37666140

[3] Liu D, Keesing JK, He P, Wang Z, Shi Y, Wang Y. 2013. The world’s largest macroalgal bloom in the Yellow Sea, China: formation and implications. Estuar Coast Shelf Sci 129:2–10. doi:10.1016/j.ecss.2013.05.021

[4] Sun Y, Liu J, Xia J, Tong Y, Li C, Zhao S, Zhuang M, Zhao X, Zhang J, He P. 2022. Research development on resource utilization of green tide algae from the Southern Yellow Sea. Energy Rep 8:295–303. doi:10.1016/j.egyr.2022.01.168

[5] Wang Y, Liu F, Liu X, Shi S, Bi Y, Moejes FW. 2019. Comparative transcriptome analysis of four co-occurring Ulva species for understanding the dominance of Ulva prolifera in the Yellow Sea green tides. J Appl Phycol 31:3303–3316. doi:10.1007/s10811-019-01810-z

[6] Smetacek V, Zingone A. 2013. Green and golden seaweed tides on the rise. Nature 504:84–88. doi:10.1038/nature12860 24305152

[7] Kidgell JT, Magnusson M, de Nys R, Glasson CRK. 2019. Ulvan: a systematic review of extraction, composition and function. Algal Res 39:101422. doi:10.1016/j.algal.2019.101422

[8] Lahaye M, Robic A. 2007. Structure and functional properties of ulvan, a polysaccharide from green seaweeds. Biomacromolecules 8:1765–1774. doi:10.1021/bm061185q 17458931

[9] Lahaye M. 1998. NMR spectroscopic characterisation of oligosaccharides from two Ulva rigida ulvan samples (Ulvales, Chlorophyta) degraded by a lyase. Carbohydr Res 314:1–12. doi:10.1016/s0008-6215(98)00293-6 10230036

[10] Paradossi G, Cavalieri F, Pizzoferrato L, Liquori AM. 1999. A physico-chemical study on the polysaccharide ulvan from hot water extraction of the macroalga Ulva. Int J Biol Macromol 25:309–315. doi:10.1016/s0141-8130(99)00049-5 10456771

[11] Qi X, Mao W, Gao Y, Chen Y, Chen Y, Zhao C, Li N, Wang C, Yan M, Lin C, Shan J. 2012. Chemical characteristic of an anticoagulant-active sulfated polysaccharide from Enteromorpha clathrata. Carbohydr Polym 90:1804–1810. doi:10.1016/j.carbpol.2012.07.077 22944450

[12] Cho M, Yang C, Kim SM, You S. 2010. Molecular characterization and biological activities of watersoluble sulfated polysaccharides from Enteromorpha prolifera. Food Sci Biotechnol 19:525–533. doi:10.1007/s10068-010-0073-3

[13] Klongklaew N, Praiboon J, Tamtin M, Srisapoome P. 2021. Chemical composition of a hot water crude extract (HWCE) from Ulva intestinalis and its potential effects on growth performance, immune responses, and resistance to white spot syndrome virus and yellowhead virus in Pacific white shrimp (Litopenaeus vannamei). Fish Shellfish Immunol 112:8–22. doi:10.1016/j.fsi.2021.02.004 33600947

[14] Fernández-Díaz C, Coste O, Malta E. 2017. Polymer chitosan nanoparticles functionalized with Ulva ohnoi extracts boost in vitro ulvan immunostimulant effect in Solea senegalensis macrophages. Algal Res 26:135–142. doi:10.1016/j.algal.2017.07.008

[15] Chen J, Zeng W, Gan J, Li Y, Pan Y, Li J, Chen H. 2021. Physicochemical properties and anti-oxidation activities of ulvan from Ulva pertusa Kjellm. Algal Res 55:102269. doi:10.1016/j.algal.2021.102269

[16] Yuan Y, Xu X, Jing C, Zou P, Zhang C, Li Y. 2018. Microwave assisted hydrothermal extraction of polysaccharides from Ulva prolifera: functional properties and bioactivities. Carbohydr Polym 181:902–910. doi:10.1016/j.carbpol.2017.11.061 29254052

[17] Shao P, Pei Y, Fang Z, Sun P. 2014. Effects of partial desulfation on antioxidant and inhibition of DLD cancer cell of Ulva fasciata polysaccharide. Int J Biol Macromol 65:307–313. doi:10.1016/j.ijbiomac.2014.01.043 24463264

[18] Sathivel A, Raghavendran HRB, Srinivasan P, Devaki T. 2008. Anti-peroxidative and anti-hyperlipidemic nature of Ulva lactuca crude polysaccharide on d-galactosamine induced hepatitis in rats. Food Chem Toxicol 46:3262–3267. doi:10.1016/j.fct.2008.07.016 18706469

[19] Li W, Wang K, Jiang N, Liu X, Wan M, Chang X, Liu D, Qi H, Liu S. 2018. Antioxidant and antihyperlipidemic activities of purified polysaccharides from Ulva pertusa. J Appl Phycol 30:2619–2627. doi:10.1007/s10811-018-1475-5

[20] Shefer S, Robin A, Chemodanov A, Lebendiker M, Bostwick R, Rasmussen L, Lishner M, Gozin M, Golberg A. 2021. Fighting SARS-CoV-2 with green seaweed Ulva sp. extract: extraction protocol predetermines crude ulvan extract anti-SARS-CoV-2 inhibition properties in in vitro Vero-E6 cells assay. PeerJ 9:e12398. doi:10.7717/peerj.12398 34820178 PMC8601053

[21] Chi Y, Zhang M, Wang X, Fu X, Guan H, Wang P. 2020. Ulvan lyase assisted structural characterization of ulvan from Ulva pertusa and its antiviral activity against vesicular stomatitis virus. Int J Biol Macromol 157:75–82. doi:10.1016/j.ijbiomac.2020.04.187 32344076

[22] Barakat KM, Ismail MM, Abou El Hassayeb HE, El Sersy NA, Elshobary ME. 2022. Chemical characterization and biological activities of ulvan extracted from Ulva fasciata (Chlorophyta). Rend Fis Acc Lincei 33:829–841. doi:10.1007/s12210-022-01103-7

[23] Tang T, Cao S, Zhu B, Li Q. 2021. Ulvan polysaccharide-degrading enzymes: an updated and comprehensive review of sources category, property, structure, and applications of ulvan lyases. Algal Res 60:102477. doi:10.1016/j.algal.2021.102477

[24] Reisky L, Préchoux A, Zühlke M-K, Bäumgen M, Robb CS, Gerlach N, Roret T, Stanetty C, Larocque R, Michel G, Song T, Markert S, Unfried F, Mihovilovic MD, Trautwein-Schult A, Becher D, Schweder T, Bornscheuer UT, Hehemann J-H. 2019. A marine bacterial enzymatic cascade degrades the algal polysaccharide ulvan. Nat Chem Biol 15:803–812. doi:10.1038/s41589-019-0311-9 31285597

[25] Nyvall Collén P, Sassi J-F, Rogniaux H, Marfaing H, Helbert W. 2011. Ulvan lyases isolated from the flavobacteria Persicivirga ulvanivorans are the first members of a new polysaccharide lyase family. J Biol Chem 286:42063–42071. doi:10.1074/jbc.M111.271825 22009751 PMC3234910

[26] Li Q, Hu F, Zhu B, Ni F, Yao Z. 2020. Insights into ulvan lyase: review of source, biochemical characteristics, structure and catalytic mechanism. Crit Rev Biotechnol 40:432–441. doi:10.1080/07388551.2020.1723486 32050804

[27] Kopel M, Helbert W, Belnik Y, Buravenkov V, Herman A, Banin E. 2016. New family of ulvan lyases identified in three isolates from the Alteromonadales order. J Biol Chem 291:5871–5878. doi:10.1074/jbc.M115.673947 26763234 PMC4786721

[28] Qin H-M, Xu P, Guo Q, Cheng X, Gao D, Sun D, Zhu Z, Lu F. 2018. Biochemical characterization of a novel ulvan lyase from Pseudoalteromonas sp. strain PLSV. RSC Adv 8:2610–2615. doi:10.1039/c7ra12294b 35541464 PMC9077492

[29] He C, Muramatsu H, Kato S-I, Ohnishi K. 2017. Characterization of an Alteromonas long-type ulvan lyase involved in the degradation of ulvan extracted from Ulva ohnoi. Biosci Biotechnol Biochem 81:2145–2151. doi:10.1080/09168451.2017.1379352 28958183

[30] Ulaganathan T, Boniecki MT, Foran E, Buravenkov V, Mizrachi N, Banin E, Helbert W, Cygler M. 2017. New ulvan-degrading polysaccharide lyase family: structure and catalytic mechanism suggests convergent evolution of active site architecture. ACS Chem Biol 12:1269–1280. doi:10.1021/acschembio.7b00126 28290654

[31] Foran E, Buravenkov V, Kopel M, Mizrahi N, Shoshani S, Helbert W, Banin E. 2017. Functional characterization of a novel “ulvan utilization loci” found in Alteromonas sp. LOR genome. Algal Res 25:39–46. doi:10.1016/j.algal.2017.04.036

[32] Konasani VR, Jin C, Karlsson NG, Albers E. 2018. A novel ulvan lyase family with broad-spectrum activity from the ulvan utilisation loci of Formosa agariphila KMM 3901. Sci Rep 8:14713. doi:10.1038/s41598-018-32922-0 30279430 PMC6168547

[33] Lombard V, Golaconda Ramulu H, Drula E, Coutinho PM, Henrissat B. 2014. The carbohydrate-active enzymes database (CAZy) in 2013. Nucleic Acids Res 42:D490–D495. doi:10.1093/nar/gkt1178 24270786 PMC3965031

[34] Konasani VR, Jin C, Karlsson NG, Albers E. 2018. Ulvan lyase from Formosa agariphila and its applicability in depolymerisation of ulvan extracted from three different Ulva species. Algal Res 36:106–114. doi:10.1016/j.algal.2018.10.016

[35] Ulaganathan T, Helbert W, Kopel M, Banin E, Cygler M. 2018. Structure-function analyses of a PL24 family ulvan lyase reveal key features and suggest its catalytic mechanism. J Biol Chem 293:4026–4036. doi:10.1074/jbc.RA117.001642 29382716 PMC5857984

[36] Ulaganathan T, Banin E, Helbert W, Cygler M. 2018. Structural and functional characterization of PL28 family ulvan lyase NLR48 from Nonlabens ulvanivorans. J Biol Chem 293:11564–11573. doi:10.1074/jbc.RA118.003659 29875159 PMC6065169

[37] Reisky L, Stanetty C, Mihovilovic MD, Schweder T, Hehemann J-H, Bornscheuer UT. 2018. Biochemical characterization of an ulvan lyase from the marine flavobacterium Formosa agariphila KMM 3901^T^. Appl Microbiol Biotechnol 102:6987–6996. doi:10.1007/s00253-018-9142-y 29948117

[38] Gao J, Du CY, Chi YZ, Zuo SQ, Ye H, Wang P. 2019. Cloning, expression, and characterization of a new PL25 family ulvan lyase from marine bacterium Alteromonas sp. A321. Mar Drugs 17:568. doi:10.3390/md17100568 31597240 PMC6836179

[39] Xu F, Dong F, Sun X-H, Cao H-Y, Fu H-H, Li C-Y, Zhang X-Y, McMinn A, Zhang Y-Z, Wang P, Chen X-L. 2021. Mechanistic insights into substrate recognition and catalysis of a new ulvan lyase of polysaccharide lyase family 24. Appl Environ Microbiol 87:00412–00421. doi:10.1128/AEM.00412-21 PMC8174760 33771786

[40] Terrapon N, Lombard V, Drula É, Lapébie P, Al-Masaudi S, Gilbert HJ, Henrissat B. 2018. PULDB: the expanded database of polysaccharide utilization loci. Nucleic Acids Res 46:D677–D683. doi:10.1093/nar/gkx1022 29088389 PMC5753385

[41] Holm L, Rosenström P. 2010. Dali server: conservation mapping in 3D. Nucleic Acids Res 38:W545–W549. doi:10.1093/nar/gkq366 20457744 PMC2896194

[42] Hashimoto W, Maruyama Y, Nakamichi Y, Mikami B, Murata K. 2014. Crystal structure of Pedobacter heparinus heparin lyase Hep III with the active site in a deep cleft. Biochemistry 53:777–786. doi:10.1021/bi4012463 24437462

[43] Zhang Q, Cao H-Y, Wei L, Lu D, Du M, Yuan M, Shi D, Chen X, Wang P, Chen X-L, Chi L, Zhang Y-Z, Li F. 2021. Discovery of exolytic heparinases and their catalytic mechanism and potential application. Nat Commun 12:1263. doi:10.1038/s41467-021-21441-8 33627653 PMC7904915

[44] Park D, Jagtap S, Nair SK. 2014. Structure of a PL17 family alginate lyase demonstrates functional similarities among exotype depolymerases. J Biol Chem 289:8645–8655. doi:10.1074/jbc.M113.531111 24478312 PMC3961687

[45] Ji S, Dix SR, Aziz AA, Sedelnikova SE, Baker PJ, Rafferty JB, Bullough PA, Tzokov SB, Agirre J, Li F-L, Rice DW. 2019. The molecular basis of endolytic activity of a multidomain alginate lyase from Defluviitalea phaphyphila, a representative of a new lyase family, PL39. J Biol Chem 294:18077–18091. doi:10.1074/jbc.RA119.010716 31624143 PMC6885643

[46] Gucwa M, Lenkiewicz J, Zheng H, Cymborowski M, Cooper DR, Murzyn K, Minor W. 2023. CMM-An enhanced platform for interactive validation of metal binding sites. Protein Sci 32:e4525. doi:10.1002/pro.4525 36464767 PMC9794025

[47] Landau M, Mayrose I, Rosenberg Y, Glaser F, Martz E, Pupko T, Ben-Tal N. 2005. ConSurf 2005: the projection of evolutionary conservation scores of residues on protein structures. Nucleic Acids Res 33:W299–W302. doi:10.1093/nar/gki370 15980475 PMC1160131

[48] Eberhardt J, Santos-Martins D, Tillack AF, Forli S. 2021. AutoDock Vina 1.2.0: new docking methods, expanded force field, and python bindings. J Chem Inf Model 61:3891–3898. doi:10.1021/acs.jcim.1c00203 34278794 PMC10683950

[49] Trott O, Olson AJ. 2010. AutoDock Vina: improving the speed and accuracy of docking with a new scoring function, efficient optimization, and multithreading. J Comput Chem 31:455–461. doi:10.1002/jcc.21334 19499576 PMC3041641

[50] Inoue A, Ojima T. 2017. Ⅲ-4. Alginate-modifying enzymes. Nippon Suisan Gakkaishi 83:827–827. doi:10.2331/suisan.WA2442-13

[51] Vernette C, Lecubin J, Sánchez P, Coordinators TO, Sunagawa S, Delmont TO, Acinas SG, Pelletier E, Hingamp P, Lescot M. 2022. The Ocean Gene Atlas v2.0: online exploration of the biogeography and phylogeny of plankton genes. Nucleic Acids Res 50:W516–W526. doi:10.1093/nar/gkac420 35687095 PMC9252727

[52] Villar E, Vannier T, Vernette C, Lescot M, Cuenca M, Alexandre A, Bachelerie P, Rosnet T, Pelletier E, Sunagawa S, Hingamp P. 2018. The Ocean Gene Atlas: exploring the biogeography of plankton genes online. Nucleic Acids Res 46:W289–W295. doi:10.1093/nar/gky376 29788376 PMC6030836

[53] Zheng J, Ge Q, Yan Y, Zhang X, Huang L, Yin Y. 2023. dbCAN3: automated carbohydrate-active enzyme and substrate annotation. Nucleic Acids Res 51:W115–W121. doi:10.1093/nar/gkad328 37125649 PMC10320055

[54] Drula E, Garron M-L, Dogan S, Lombard V, Henrissat B, Terrapon N. 2022. The carbohydrate-active enzyme database: functions and literature. Nucleic Acids Res 50:D571–D577. doi:10.1093/nar/gkab1045 34850161 PMC8728194

[55] Letunic I, Bork P. 2024. Interactive Tree of Life (iTOL) v6: recent updates to the phylogenetic tree display and annotation tool. Nucleic Acids Res 52:W78–W82. doi:10.1093/nar/gkae268 38613393 PMC11223838

[56] Agirre J, Atanasova M, Bagdonas H, Ballard CB, Baslé A, Beilsten-Edmands J, Borges RJ, Brown DG, Burgos-Mármol JJ, Berrisford JM, et al.. 2023. The CCP4 suite: integrative software for macromolecular crystallography. Acta Crystallogr D Struct Biol 79:449–461. doi:10.1107/S2059798323003595 37259835 PMC10233625

[57] Mirdita M, Schütze K, Moriwaki Y, Heo L, Ovchinnikov S, Steinegger M. 2022. ColabFold: making protein folding accessible to all. Nat Methods 19:679–682. doi:10.1038/s41592-022-01488-1 35637307 PMC9184281

[58] Adams PD, Grosse-Kunstleve RW, Hung LW, Ioerger TR, McCoy AJ, Moriarty NW, Read RJ, Sacchettini JC, Sauter NK, Terwilliger TC. 2002. PHENIX: building new software for automated crystallographic structure determination. Acta Crystallogr D Biol Crystallogr 58:1948–1954. doi:10.1107/s0907444902016657 12393927

[59] Emsley P, Cowtan K. 2004. Coot: model-building tools for molecular graphics. Acta Crystallogr D Struct Biol 60:2126–2132. doi:10.1107/S0907444904019158 15572765

