# Supplementary material for: Structural and functional insights into Uly1040, an ulvan lyase from polysaccharide lyase family 40
Source: Appl Environ Microbiol. 2026 Jan 14;92(2):e02101-25. doi: 10.1128/aem.02101-25 (PMC12915356; doi:10.1128/aem.02101-25)
Supplement: Supplemental material — Figures S1 to S5; Tables S1 to S4. [file aem.02101-25-s0001.docx]

**Supporting information for:**

**Structural and functional insights into Uly1040, an ulvan lyase from polysaccharide lyase family 40**

**This PDF file includes:**

Supporting Figure 1-5

Supporting Figure Legends

Supporting Table 1-4


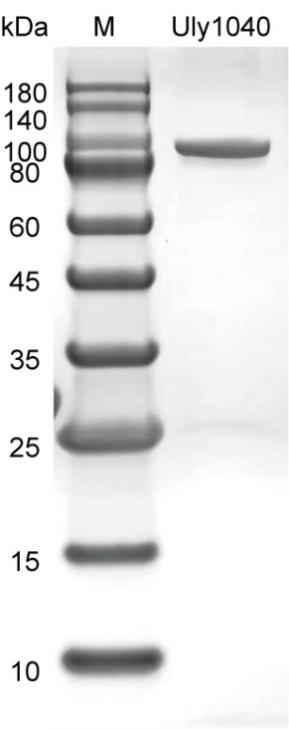


**Figure S1. SDS-PAGE analysis of the purified Uly1040.**


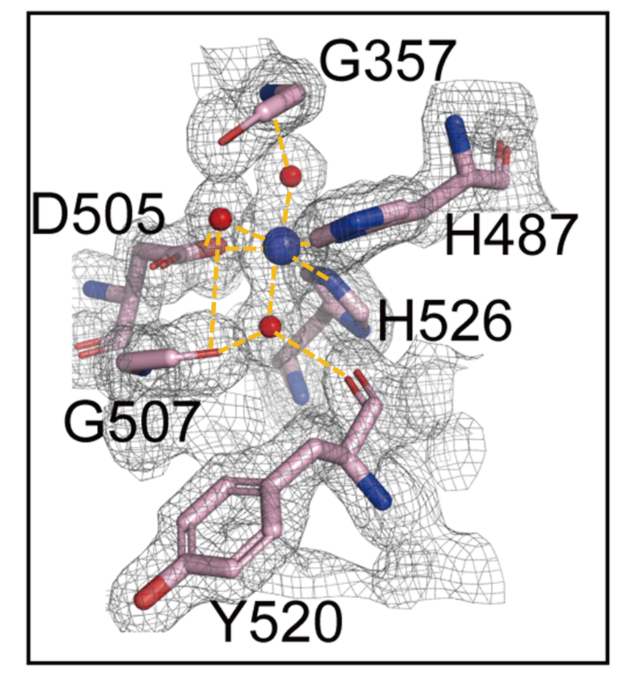


**Figure S2. Mn^2+^ binding site in Uly1040.** The Mn^2+^ ion is coordinated by His487, Asp505, and His526, as well as by water molecules that form hydrogen bonds to Gly357, Gly507, Asp505 and Tyr520. The gray mesh represents the |*F_o_*| - |*F_c_*| omit electron density map,**contoured at 1.2 σ**.


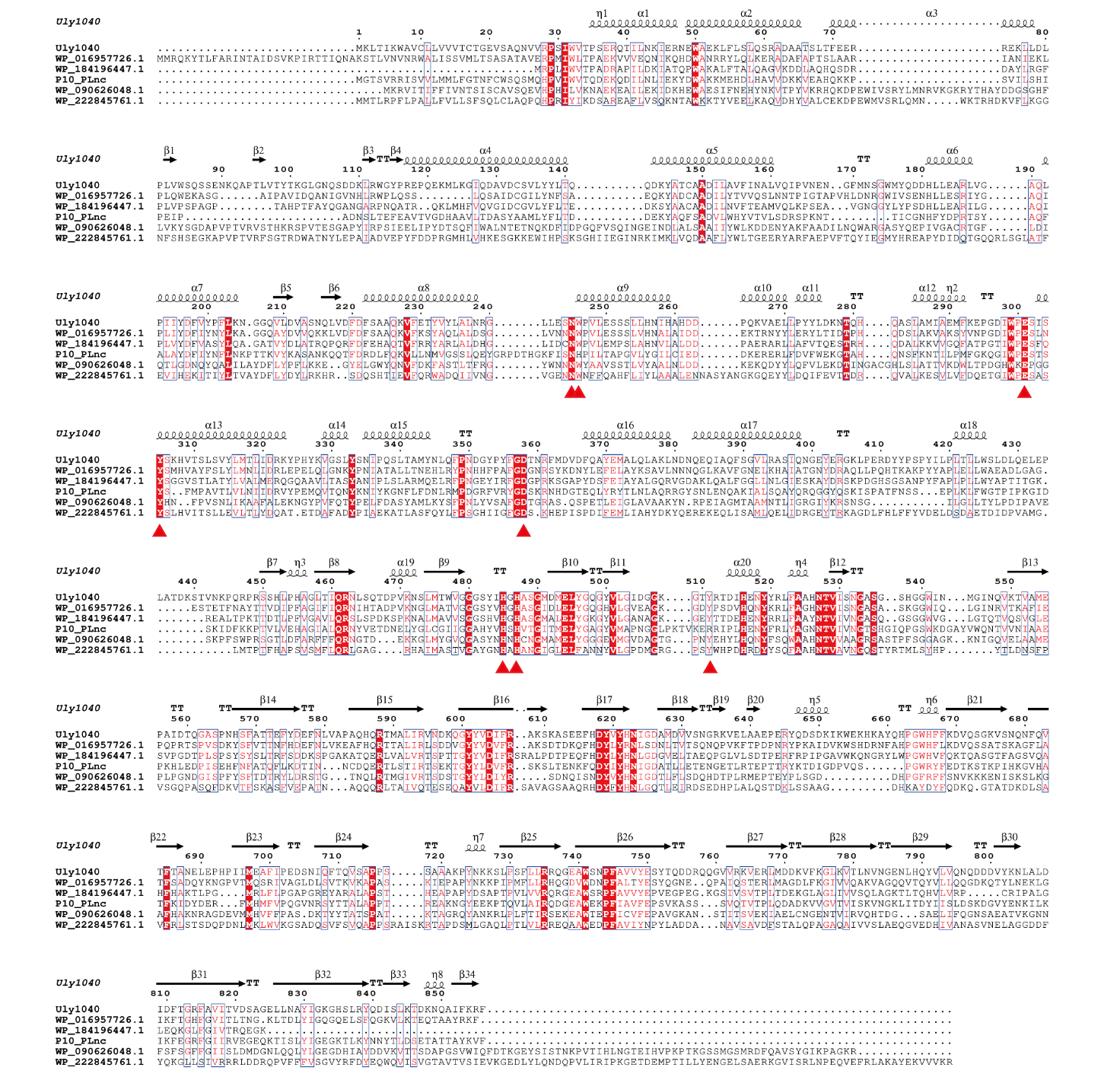


**Figure S3. Sequence alignment of Uly1040 and PL40 homologs.** Identical and similar amino acid residues are shaded. The red triangle indicates conserved amino acids near the catalytic cavity.


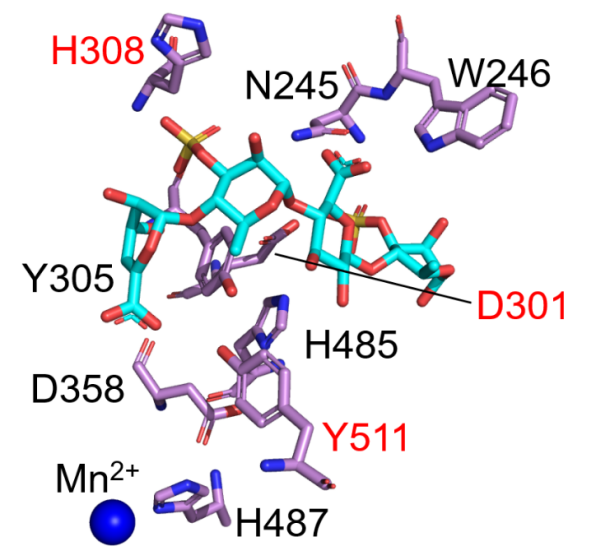


**Figure S4. Putative polar residues in the catalytic cavity involved in substrate recognition.**


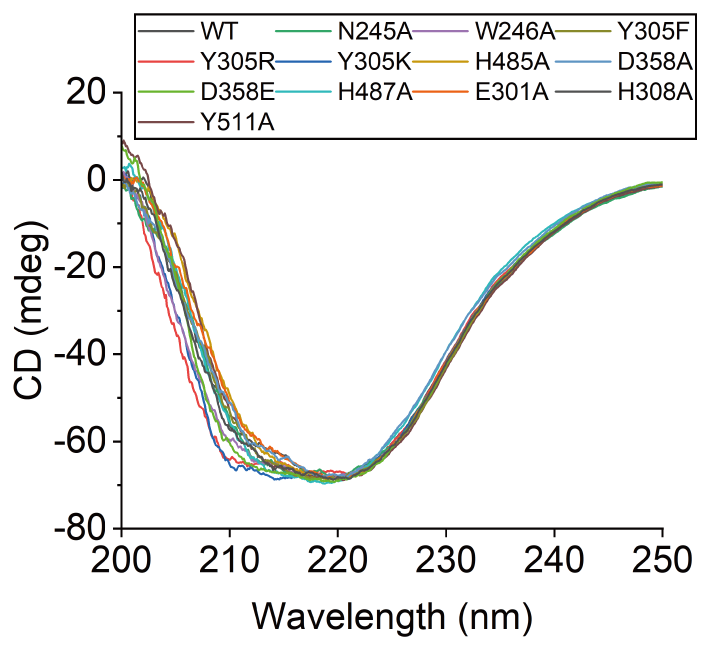


**Figure S5. Circular dichroism (CD) spectra of WT Uly1040 and its mutants.** CD spectra of the proteins at a final concentration of approximately 10 μM were collected from 200 to 250 nm.

**Table S1. Structural homologs of Uly1040 analyzed by the Dali server.**

| **Protein** | **PDB code** | **PL family** | **Z score** | **RMSD** | **LALI (Å) ^a^** | **Function** |
| --- | --- | --- | --- | --- | --- | --- |
| Alg17C | 4NEI | PL17 | 33.5 | 3.8 | 624 | Alginate lyase |
| Dp0100 | 6JPN | PL39 | 27.7 | 3.9 | 616 | Alginate lyase |
| Phep_3797 | 4MMI | PL12 | 25.3 | 4.5 | 529 | Heparinase III |
| BiexoHep | 6LJA | PL15 | 24.6 | 4.0 | 581 | Heparinase II/ III |

Entries are ranked by Dali Z-score (descending).

^a^ Total number of the equivalenced residues is shown.

**Table S2. Inductively coupled plasma optical emission spectrometry (ICP-OES) analysis of Uly1040.**

| **Element** | **Mn** | **Fe** | **Mg** | **Ca** | **Al** | **Ni** | **K** | **Zn** | **Cu** | **Ba** |
| --- | --- | --- | --- | --- | --- | --- | --- | --- | --- | --- |
| Number of atoms per subunit | 0.33 | 0.32 | 0.22 | 0.22 | 0.12 | 0.01 | 0.07 | 0.05 | 0.00 | 0.00 |

**Table S3. Validation of the Mn^2+^ binding site by the CheckMyMetal server.**

| **Metal** | **Occupancy** | **B factor (env.)^a^** | **Ligands** | **Valence^b^** | **nVECSUM^c^** | **Geometry^d^** | **gRMSD (°)^e^** | **Vacancy^f^** | **Bidentate^g^** |
| --- | --- | --- | --- | --- | --- | --- | --- | --- | --- |
| Mn | 1.0 | 16.8 (16.8) | O_4_N_2_ | 2.1 | 0.15 | Octahedral | 6.1° | 0 | 0.0 |

The color scheme indicates the validation outcome: gray (not applicable), yellow (borderline), and green (acceptable).

^a^ Metal ion B factor, with valence-weighted environmental average B factor in parenthesis.

^b^ Summation of bond valence values for an ion binding site. Valence accounts for metal-ligand distances.

^c^ Summation of ligand vectors, weighted by bond valence values and normalized by overall valence.

Increase when the coordination sphere is not symmetrical due to incompleteness.

^d^ Arrangement of ligands around the ion, as defined by the NEIGHBORHOOD algorithm.

^e^ R.M.S. Deviation of observed geometry angles (L-M-L angles) compared to ideal geometry, in degrees.

^f^ Percentage of unoccupied sites in the coordination sphere for the given geometry.

^g^ Number of residues that form a bidentate interaction instead of being considered as multiple ligands.

**Table S4. Effects of metal ions and EDTA on Uly1040 enzyme activity.**

| **Compound** | **Relative activity (%)^a^** | |
| --- | --- | --- |
|  | **0.25 mM** | **1 mM** |
| EDTA | 87.31±1.18 | 97.59±1.33 |
| Ca^2+^ | 101.84±1.79 | 86.28±3.13 |
| Mg^2+^ | 99.95±4.36 | 97.47±2.14 |
| Fe^3+^ | 90.39±2.56 | 42.32±3.53 |
| K^+^ | 88.00±6.14 | 84.44±3.52 |
| Mn^2+^ | 80.74±4.62 | 85.47±2.23 |
| Zn^2+^ | 83.65±2.28 | 26.94±0.51 |
| Cu^2+^ | 85.91±2.28 | 10.11±2.00 |

^a^ The activity of WT Uly1040 was defined as 100%.

All experiments were repeated three times.
